# Supplementary material for: Providing normative information increases intentions to accept a COVID-19 vaccine
Source: Nat Commun. 2023 Jan 9;14:126. doi: 10.1038/s41467-022-35052-4 (PMC9828376; doi:10.1038/s41467-022-35052-4)
Supplement: Supplementary file 1 — Supplementary Information [file 41467_2022_35052_MOESM1_ESM.pdf]

Supplementary Information
Providing normative information increases intentions to accept a COVID-19 vaccine

Alex Moehring[1,2], **Avinash Collis**[3,†], **Kiran Garimella**[4,†], **M. Amin Rahimian**[2,5,†], **Sinan Aral**[1,2,6], and **Dean Eckles**[1,2,6,‡]

[1]Sloan School of Management, Massachusetts Institute of Technology, Cambridge, MA, USA; [2]MIT Initiative on the Digital Economy, Massachusetts Institute of Technology, Cambridge, MA, USA; [3]McCombs School of Business, The University of Texas at Austin, Austin, TX, USA; [4]School of Communication and Information, Rutgers University, New Brunswick, NJ, USA; [5]Department of Industrial Engineering, University of Pittsburgh, Pittsburgh, PA, USA; [6]Institute for Data, Systems, and Society, Massachusetts Institute of Technology, Cambridge, MA, USA

[†] A.C., K.G., and M.A.R. contributed equally to this work and are listed alphabetically.
[‡] To whom correspondence should be addressed. E-mail: eckles@mit.edu

Contents

Experiment overview 3

Variables of interest 6

Randomization checks 7

Effects on beliefs about descriptive norms 13

Effects on intentions 14

Norm–intention correlations 28

Country-level validation of survey data 29

Intention to behavior correlation 29

Supplementary Note 1. Experiment overview

Figure S1 shows the recruitment materials displayed on Facebook. Figure S2 outlines the basic experimental design. Figure S3 shows the various norms shown to participants. There is variation over time as these numbers were updated to have the most recent data throughout the experiment.

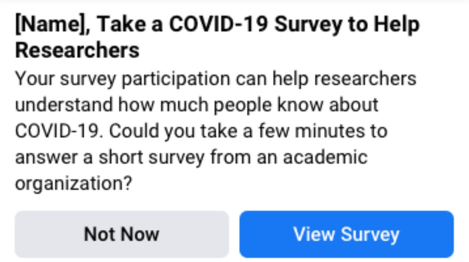

(a) Facebook Recruitment Message

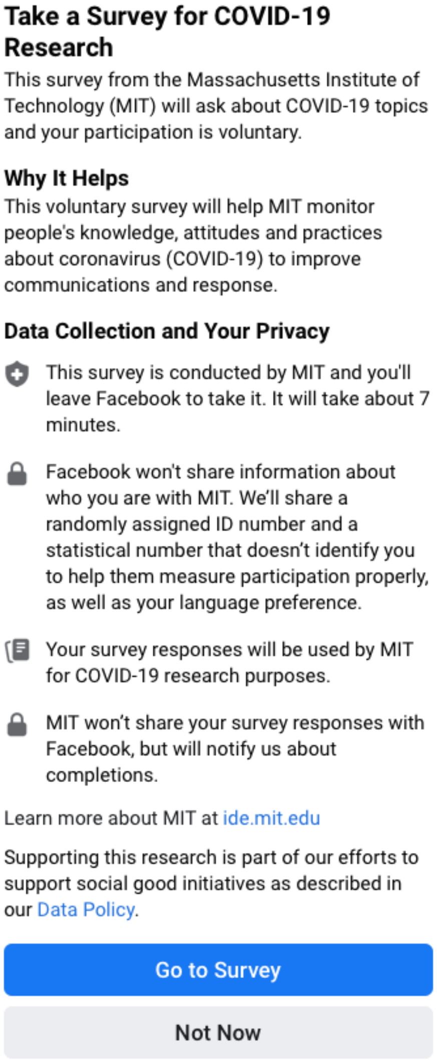

(b) Facebook Interstitial

Fig. S1. Facebook Promotion and Interstitial.

(a) Initial recruitment message shown at the top of a Facebook user's home page. (b) Interstitial shown to users who clicked View Survey from (a) that described the survey and how the data would be used in detail.

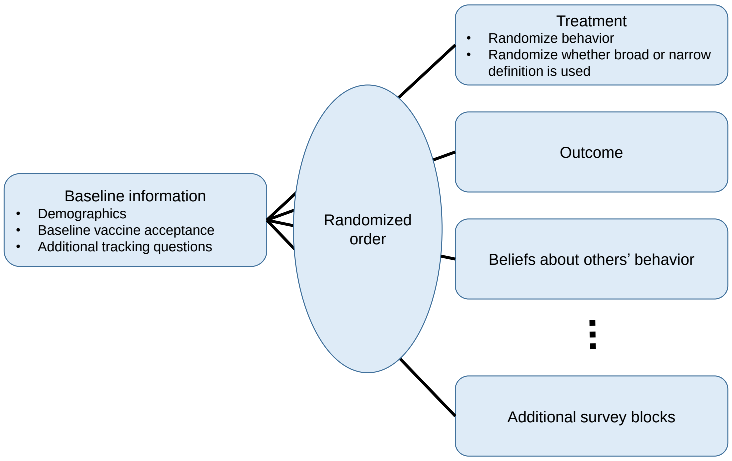

Fig. S2. Experiment Flow

Illustration of the flow of a respondent through the survey. First, they are presented with tracking and demographic questions. They then enter a randomized portion where blocks are in random order. This includes the treatment, outcome, and many of the baseline covariates included in regressions for precision. Recall all covariates used in analysis are only used if they are pre-treatment and outcome.

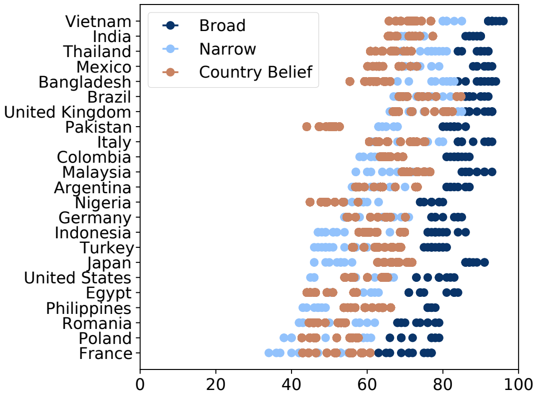

(a) Vaccine Treatments

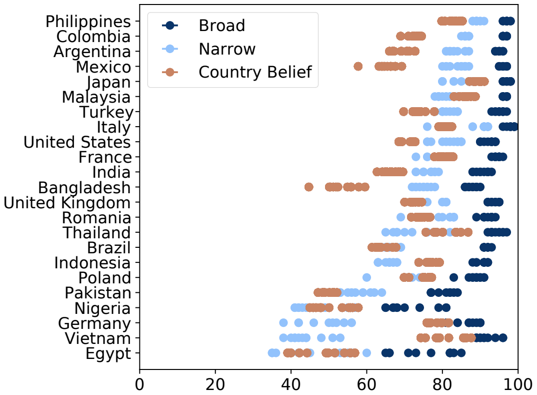

(b) Mask Treatments

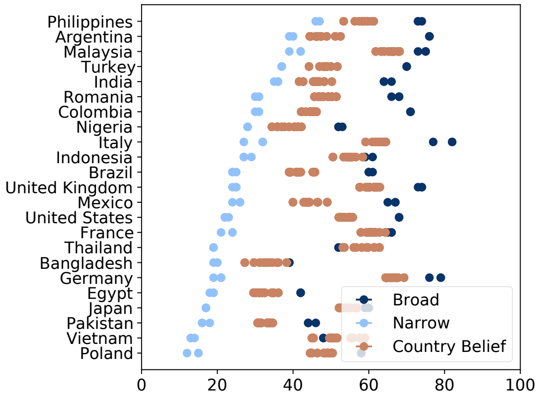

(c) Distancing Treatments

Fig. S3. Treatment Variation

For each behavior ((a) Vaccine, (b) Mask wearing, (c) Physical distancing), we plot the information provided to participants based on the broad and narrow definitions of compliance. The treatments were updated every two weeks as new waves of data were included. The points labeled “country belief” display the weighted average belief in a country of how many people out of 100 practice (or will accept, for vaccines) each behavior.

Supplementary Note 2. Variables of interest

As in our pre-analysis plan, the following variables are used in our analysis:

1. Outcomes
   (a) Over the next two weeks, how likely are you to wear a mask when in public? [Always, Almost always, When convenient, Rarely, Never]
   (b) Over the next two weeks, how likely are you to maintain a distance of at least 1 meter from others when in public? [Always, Almost always, When convenient, Rarely, Never]
   (c) If a vaccine against COVID-19 infection is available in the market, would you take it? [Yes, definitely, Probably, Unsure, Probably not, No, definitely not]
2. Mediators & Covariates
   (a) Baseline outcomes. These questions are similar to the outcome questions. Only the vaccine question always appears before the treatment in all cases; the others are in a randomized order. Thus, for use of the other covariates for increasing precision, mean imputation is required.
      - Masks. How often are you able to wear a mask or face covering when you are in public? How effective is wearing a face mask for preventing the spread of COVID-19?
      - Distancing: How often are you able to stay at least 1 meter away from people not in your household? How important do you think physical distancing is for slowing the spread of COVID-19?
      - Vaccine: If a vaccine for COVID-19 becomes available, would you choose to get vaccinated? This will be coded as binary indicators for the possible outcomes, grouping missing outcomes with “Don’t know”.

   (b) Beliefs about norms. These questions will be randomized to be shown before the treatment for some respondents and after treatment for other respondents. This will allow us to study heterogeneity in baseline beliefs, as well as ensure our randomization does impact beliefs.
      - Masks: Out of 100 people in your community, how many do you think do the following when they go out in public? Wear a mask or face covering.

- Distancing: Out of 100 people in your community, how many do you think do the following when they go out in public? Maintain a distance of at least 1 meter from others.
- Vaccine: Out of 100 people in your community, how many do you think would take a COVID-19 vaccine if it were made available?

3. Additional covariates used to check balance

   (a) Indicators if respondents received news from the following sources and mediums: online sources, messaging apps, newspapers, television, radio, local health workers, scientists, the World Health Organization, politicians, journalists, and peers.

   (b) Indicators if respondents trusted news from the same sources and mediums as above.

   (c) Indicators if respondents reported engaging the following behaviors: wearing a face mask, taking herbal supplements, using homeopathic remedies, getting the flu vaccine, eating garlic, cleaning surfaces, using antibiotics, isolation, hand washing, covering their mouth when they cough, avoid sick individuals, maintain a distance from other, avoid touching their face, and caution opening mail.

   (d) Indicators if respondents reported being willing to attend restaurants, parks and beaches, retail shops, schools, performances and sporting events, places of employment, places of worship, and health care facilities.

When used in analysis, we require all covariates to be before both treatment and outcome. As the survey contains randomized order for these questions, this ensures that the distribution of question order is the same across treated and control groups and removes any imbalance created by differential attrition. Missing values are imputed at their (weighted) mean.

Supplementary Note 3. Randomization checks

Table S1 presents results of a test that the treatment and control shares were equal to 50% as expected. While the final dataset does have some evidence of imbalance that could be caused by differential attrition, the "robust" dataset (described in Supplementary Note 5.1) is well balanced and the treatment is balanced across the three behaviors information could be provided about (Table S2). According to our pre-registered analysis plan, in the presence of evidence of differential attrition, we make use of additional analyses that use the information about other behaviors as an alternative control group throughout this supplement.

Table S1. Randomization Tests

	Treated Share	Treated CI	Control Share	Control CI	p-val
Full	0.501	[0.500, 0.503]	0.499	[0.497, 0.500]	0.011
Final	0.499	[0.497, 0.500]	0.501	[0.500, 0.503]	0.081
Robust	0.499	[0.497, 0.501]	0.501	[0.499, 0.503]	0.176

The results of a two-sided test that the treated share and control shares equal 50%. The first row uses intent-to-treat on the full set of eligible respondents, the second row uses the final data set after conditioning on eligibility and completing the survey, and the third row uses the subset of responses in the final dataset that have at least one block between treatment and outcome. The CI columns are 95% confidence intervals.

Table S2. Randomization Tests

		Vaccine	Masks	Distancing
Final	Share	0.576	0.336	0.088
	CI	[0.574, 0.578]	[0.334, 0.338]	[0.087, 0.090]
	p-value	0.215	0.218	0.441
Robust	Share	0.577	0.336	0.086
	CI	[0.575, 0.580]	[0.334, 0.339]	[0.085, 0.088]
	p-value	0.210	0.113	0.519

The p-values of a two-sided test that each behavior was shown the expected number of times. This reports the results of a joint test that each period share was equal to the expected. For waves 9-12, each behavior was shown 1/3 of the time and for waves 12 on the vaccine treatments were shown to 2/3 of respondents and the mask treatments were shown to 1/3 of respondents. This table cannot include the full dataset intent-to-treat analysis because the behavior randomization occurred when the treatment was shown. The CI rows are 95% confidence intervals.

In addition, baseline covariates measured before both treatment and the outcome are balanced across treatment and control groups (Table S3). The covariates are also balanced in the final analysis dataset (Table S4) and within treated users across the three possible treatment behaviors (Table S5). As a result of the large number of covariates available in the survey, these tables only include a subset of possible covariates. In Figure S4 we plot ordered p-values for the balance tests described in Tables S3, S4, and S5. In addition to the p-value from the tables, Figure S4 also includes p-values from similar balance checks on responses to questions that contain multiple possible answers. The additional covariates included are responses to the questions on news sources, mediums, and trust, preventative measures taken, and locations that are open. Full text of these questions are described in detail in Supplementary Note 2.

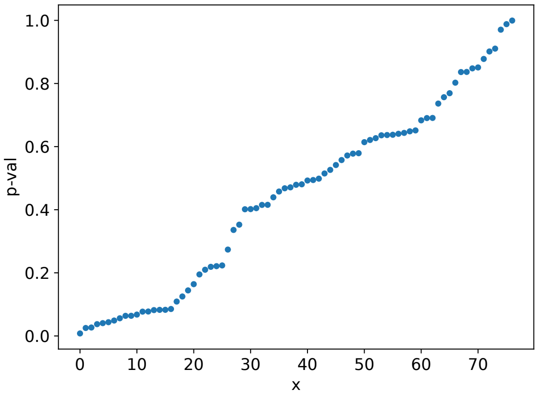

(a) Balance Tests: Intent-to-treat

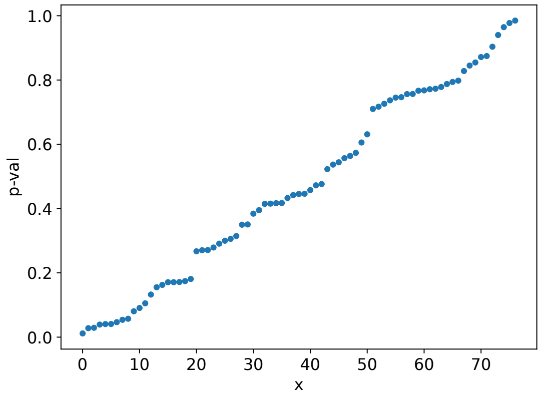

(b) Balance Tests: Final Sample

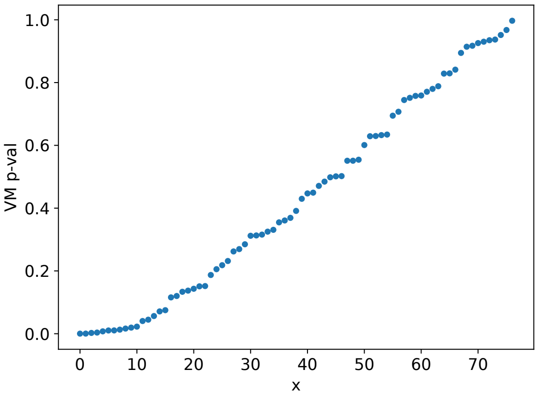

(c) Balance Tests: Vaccine vs Mask Treatments

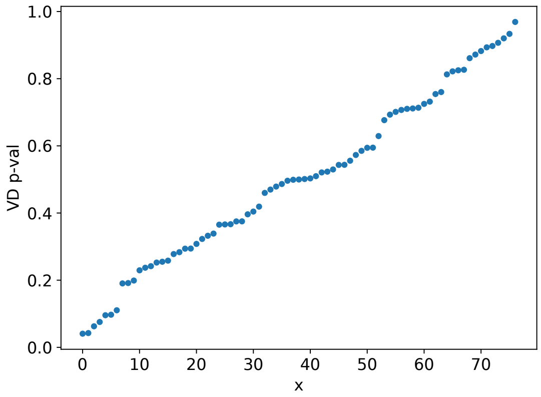

(d) Balance Tests: Vaccine vs Dist Treatments

Fig. S4. Balance Test p-Values

Ordered p-values for the (two-sided) balance tests described in Tables S3, S4, and S5 sorted in ascending order. All available pre-treatment covariates are included, which results in 76 tests. This includes all covariates reported in Table S3 in addition to questions that contain multiple responses. Full text of these questions are described in detail in (1) and are summarized in Supplementary Note 2 under additional covariates used to check balance. (a) Balance checks for intent-to-treat sample, (b) balance checks for the final sample, (c) balance checks comparing vaccine and mask treated groups, (d) balance checks comparing vaccine and physical distancing treated groups. There are no corrections applied for multiple comparisons.

Table S3. Balance Tests: Intent-to-treat

	p-val	Control	Control CI	Treated	Treated CI
age	0.223	2.587	[2.583, 2.592]	2.583	[2.578, 2.588]
gender	0.401	1.441	[1.439, 1.442]	1.440	[1.438, 1.441]
education	0.468	2.781	[2.778, 2.783]	2.779	[2.777, 2.782]
own health	0.068	2.410	[2.407, 2.413]	2.414	[2.411, 2.417]
vaccine accept	0.848	1.491	[1.488, 1.493]	1.491	[1.489, 1.493]
knowledge existing treatments	0.210	0.218	[0.217, 0.220]	0.219	[0.218, 0.220]
info exposure past week	0.439	2.300	[2.297, 2.302]	2.301	[2.298, 2.304]
info exposure more less wanted	0.614	2.387	[2.383, 2.390]	2.386	[2.382, 2.389]
know positive case	0.405	1.281	[1.278, 1.284]	1.279	[1.276, 1.282]
prevention mask	0.999	3.607	[3.603, 3.611]	3.607	[3.602, 3.611]
prevention distancing	0.769	2.669	[2.662, 2.675]	2.670	[2.664, 2.676]
prevention hand washing	0.526	3.299	[3.295, 3.304]	3.297	[3.292, 3.302]
effect mask	0.641	2.983	[2.977, 2.988]	2.981	[2.976, 2.987]
effect hand washing	0.195	2.996	[2.991, 3.001]	2.991	[2.986, 2.996]
country management	0.082	1.831	[1.824, 1.838]	1.822	[1.815, 1.829]
community management	0.878	1.929	[1.922, 1.935]	1.928	[1.922, 1.934]
community action importance	0.498	3.354	[3.349, 3.359]	3.352	[3.347, 3.356]
community action norms	0.458	2.737	[2.731, 2.744]	2.734	[2.728, 2.740]
distancing importance	0.803	3.112	[3.106, 3.117]	3.111	[3.105, 3.116]
norms dist	0.221	49.008	[48.831, 49.186]	49.162	[48.986, 49.339]
norms masks	0.648	71.800	[71.631, 71.969]	71.852	[71.683, 72.021]
norms vaccine	0.837	61.939	[61.768, 62.110]	61.917	[61.747, 62.086]
risk community	0.164	2.541	[2.531, 2.550]	2.531	[2.521, 2.540]
risk infection	0.638	2.165	[2.155, 2.175]	2.168	[2.158, 2.178]
control infection	0.515	1.879	[1.867, 1.891]	1.873	[1.861, 1.885]
infection severity	0.083	1.272	[1.265, 1.278]	1.264	[1.258, 1.270]
employed 2020	0.542	0.725	[0.721, 0.729]	0.727	[0.723, 0.731]

Pre-treatment covariate means and 95% confidence intervals for all respondents who were eligible for treatment in both the treatment and control groups along with the p-value for the two-sided test of the null that the means are equal. For each covariate, only responses where the covariate is not missing and occurs before both treatment and control are included. To account for changes to the sampling frequencies, these p-values are from the coefficient on the intent-to-treat term in a regression of the covariate on treatment, period, and centered interactions between treatment and period. As we do not have weights for all respondents, this is an unweighted regression. There are no corrections for multiple comparisons.

Table S4. Balance Tests: Final Dataset

	p-val	Control	Control CI	Treated	Treated CI
age	0.855	2.697	[2.691, 2.703]	2.696	[2.691, 2.703]
gender	0.757	1.442	[1.440, 1.444]	1.441	[1.440, 1.444]
education	0.446	2.826	[2.823, 2.829]	2.826	[2.823, 2.829]
own health	0.828	2.394	[2.390, 2.397]	2.398	[2.390, 2.397]
vaccine accept	0.710	1.510	[1.507, 1.513]	1.510	[1.507, 1.513]
knowledge existing treatments	0.417	0.213	[0.211, 0.214]	0.211	[0.211, 0.214]
info exposure past week	0.417	2.367	[2.364, 2.370]	2.371	[2.364, 2.370]
info exposure more less wanted	0.875	2.410	[2.406, 2.415]	2.409	[2.406, 2.415]
know positive case	0.029	1.329	[1.326, 1.333]	1.325	[1.326, 1.333]
prevention mask	0.181	3.640	[3.635, 3.645]	3.643	[3.635, 3.645]
prevention distancing	0.132	2.709	[2.702, 2.717]	2.716	[2.702, 2.717]
prevention hand washing	0.445	3.333	[3.327, 3.338]	3.335	[3.327, 3.338]
effect mask	0.155	2.996	[2.989, 3.003]	2.990	[2.989, 3.003]
effect hand washing	0.315	3.014	[3.008, 3.020]	3.011	[3.008, 3.020]
country management	0.537	1.796	[1.788, 1.804]	1.782	[1.788, 1.804]
community management	0.964	1.904	[1.897, 1.912]	1.900	[1.897, 1.912]
community action importance	0.747	3.371	[3.365, 3.376]	3.369	[3.365, 3.376]
community action norms	0.717	2.712	[2.705, 2.720]	2.705	[2.705, 2.720]
distancing importance	0.279	3.150	[3.143, 3.156]	3.149	[3.143, 3.156]
norms dist	0.028	49.517	[49.307, 49.727]	49.797	[49.307, 49.727]
norms masks	0.041	72.605	[72.408, 72.803]	72.918	[72.408, 72.803]
norms vaccine	0.871	62.591	[62.392, 62.789]	62.572	[62.392, 62.789]
risk community	0.163	2.564	[2.552, 2.575]	2.544	[2.552, 2.575]
risk infection	0.756	2.205	[2.194, 2.217]	2.211	[2.194, 2.217]
control infection	0.557	1.885	[1.870, 1.899]	1.874	[1.870, 1.899]
infection severity	0.011	1.269	[1.262, 1.276]	1.257	[1.262, 1.276]
employed 2020	0.415	0.725	[0.721, 0.730]	0.728	[0.721, 0.730]

Pre-treatment covariate means and 95% confidence intervals for all respondents who were eligible for treatment, completed the entire survey, and received a full survey completion weight in both the treatment and control groups along with their standard errors in parenthesis and the p-value for the two-sided test of the null that the means are equal. For each covariate, only responses where the covariate is not missing and occurs before both treatment and control are included. To account for changes to the sampling frequencies, these p-values are from the coefficient on the treatment term in a regression of the covariate on treatment, period, and centered interactions between treatment and period. This is a weighted regression using full completion survey weights. There are no corrections for multiple comparisons.

Table S5. Balance Tests Between Treatments: Final Dataset

	VD p-val	VM p-val	Vaccine	Masks	Dist	Vaccine CI	Masks CI	Dist CI
age	0.543	0.011	2.716	2.684	2.611	[2.708, 2.724]	[2.673, 2.694]	[2.591, 2.631]
gender	0.229	0.630	1.440	1.442	1.445	[1.437, 1.443]	[1.438, 1.445]	[1.438, 1.452]
education	0.861	0.313	2.825	2.826	2.839	[2.820, 2.829]	[2.820, 2.831]	[2.828, 2.849]
own health	0.278	0.629	2.399	2.401	2.386	[2.394, 2.404]	[2.394, 2.408]	[2.373, 2.399]
vaccine accept	0.419	0.000	1.524	1.505	1.442	[1.520, 1.528]	[1.499, 1.510]	[1.432, 1.453]
knowledge existing treatments	0.308	0.926	0.159	0.209	0.555	[0.157, 0.161]	[0.207, 0.212]	[0.549, 0.562]
info exposure past week	0.294	0.633	2.375	2.369	2.354	[2.370, 2.379]	[2.363, 2.374]	[2.342, 2.365]
info exposure more less wanted	0.339	0.361	2.420	2.404	2.355	[2.415, 2.426]	[2.397, 2.412]	[2.340, 2.370]
know positive case	0.825	0.894	1.339	1.326	1.233	[1.334, 1.344]	[1.320, 1.332]	[1.220, 1.246]
prevention mask	0.920	0.391	3.649	3.640	3.609	[3.642, 3.656]	[3.632, 3.649]	[3.591, 3.627]
prevention distancing	0.479	0.551	2.723	2.711	2.687	[2.713, 2.733]	[2.698, 2.724]	[2.662, 2.713]
prevention hand washing	0.500	0.134	3.339	3.331	3.326	[3.332, 3.346]	[3.322, 3.341]	[3.307, 3.346]
effect mask	0.366	0.744	2.998	2.991	2.933	[2.989, 3.007]	[2.980, 3.003]	[2.909, 2.956]
effect hand washing	0.897	0.232	3.016	3.003	3.013	[3.008, 3.024]	[2.992, 3.014]	[2.992, 3.034]
country management	0.375	0.143	1.788	1.775	1.765	[1.777, 1.800]	[1.761, 1.790]	[1.736, 1.794]
community management	0.544	0.013	1.912	1.885	1.880	[1.902, 1.923]	[1.872, 1.899]	[1.853, 1.907]
community action importance	0.710	0.751	3.370	3.369	3.367	[3.362, 3.378]	[3.359, 3.379]	[3.347, 3.387]
community action norms	0.503	0.695	2.711	2.704	2.679	[2.701, 2.720]	[2.691, 2.716]	[2.653, 2.704]
distancing importance	0.882	0.841	3.150	3.148	3.149	[3.141, 3.159]	[3.136, 3.160]	[3.125, 3.173]
norms dist	0.523	0.917	49.914	49.721	49.237	[49.640, 50.187]	[49.359, 50.084]	[48.490, 49.984]
norms masks	0.693	0.447	73.143	72.896	71.308	[72.887, 73.400]	[72.554, 73.238]	[70.587, 72.029]
norms vaccine	0.521	0.829	62.837	62.512	60.816	[62.578, 63.095]	[62.172, 62.853]	[60.085, 61.548]
risk community	0.813	0.331	2.547	2.545	2.515	[2.532, 2.563]	[2.525, 2.565]	[2.477, 2.553]
risk infection	0.460	0.771	2.218	2.208	2.179	[2.203, 2.234]	[2.188, 2.228]	[2.140, 2.218]
control infection	0.242	0.498	1.880	1.872	1.849	[1.860, 1.899]	[1.847, 1.897]	[1.802, 1.897]
infection severity	0.323	0.554	1.255	1.258	1.264	[1.246, 1.265]	[1.245, 1.271]	[1.240, 1.287]
employed 2020	0.707	0.152	0.731	0.722	0.733	[0.725, 0.738]	[0.714, 0.731]	[0.717, 0.749]

Pre-treatment covariate means and 95% confidence intervals for all respondents who were treated, completed the entire survey, and received a full survey completion weight along with their standard errors in parenthesis and the p-value for the two-sided test of the null that the means between treatment groups are equal. For each covariate, only responses where the covariate is not missing and occurs before both treatment and control are included. To account for changes to the sampling frequencies, these p-values are from the coefficient on the treatment behavior terms in a regression of the covariate on treatment behavior, period, and centered interactions between treatment behavior and period. This is a weighted regression using full completion survey weights. There are no corrections for multiple comparisons.

Supplementary Note 4. Effects on beliefs about descriptive norms

Figure S5 plots coefficients on treatment from a regression of survey norms on treatment status, including centered covariates and interactions as described in the pre-analysis plan. Figure S5a compares treatment and control respondents and Figure S5b conditions on treated individuals and then uses individuals who received an information treatment for a different behavior as control.

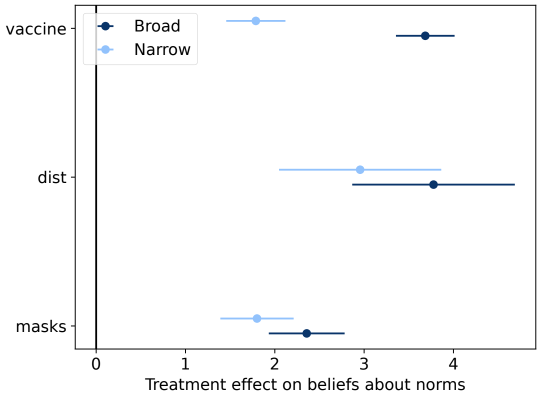

(a) Treatment vs. Control

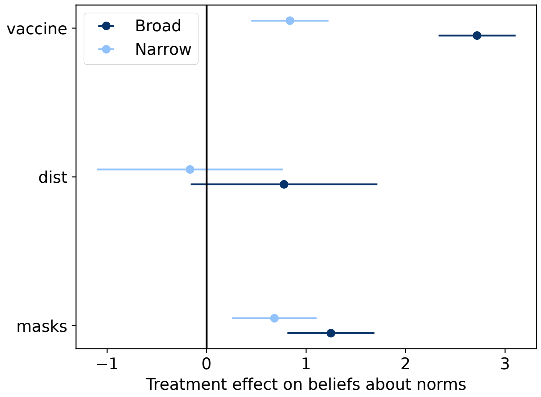

(b) Treatment vs. Other Behavior Treatment

Fig. S5. Effects on beliefs about descriptive norms

Graphical illustration of estimates displayed in Table S6. (a) Effect of treatment relative to control group. (b) Effect of treatment relative to treated groups for a different preventative behavior. Error bars are 95% confidence intervals. The exact number of responses are displayed in Table S6.

Table S6. Effects on beliefs about descriptive norms, for primary and alternative definitions of the control group

	(1)	(2)	(3)	(4)	(5)	(6)
Broad Treatment	1.250	0.778	2.719	2.357	3.777	3.685
	(0.223)	(0.479)	(0.198)	(0.217)	(0.464)	(0.168)
	[0.812 , 1.687]	[-0.160 , 1.717]	[2.331 , 3.106]	[1.933 , 2.782]	[2.867 , 4.688]	[3.356 , 4.013]
	p=2.2E-08	p=0.104	p=4.8E-43	p=1.4E-27	p=4.3E-16	p=3.9E-107
Narrow Treatment	0.682	-0.167	0.838	1.801	2.955	1.788
	(0.217)	(0.477)	(0.198)	(0.209)	(0.463)	(0.169)
	[0.257 , 1.106]	[-1.102 , 0.769]	[0.450 , 1.226]	[1.390 , 2.211]	[2.047 , 3.864]	[1.456 , 2.119]
	p=0.002	p=0.727	p=2.3E-05	p=8.3E-18	p=1.8E-10	p=4.2E-26
Control: Other Treatment	X	X	X			
Behavior	masks	dist	vaccine	masks	dist	vaccine
Number Controls	149458	34002	91217	229715	52724	225615
Number Treated	75125	17354	130389	75125	17354	130389
Observations	224,583	51,356	221,606	304,840	70,078	356,004
R^2	0.170	0.145	0.206	0.182	0.157	0.210
Adjusted R^2	0.170	0.143	0.206	0.182	0.156	0.210
Residual Std. Error	25.040	27.046	24.162	25.445	27.085	24.566
F Statistic	130.581	46.184	191.547	201.909	64.198	329.570

Estimates of equation 1 with beliefs about descriptive norms as outcomes. All p-values are from two-sided test that coefficient is equal to zero, standard errors are in parentheses, and 95% confidence intervals are in square brackets.

Supplementary Note 5. Effects on intentions

Figure S6 displays regression coefficients for the primary analysis described in the methods section. Figure S6a uses respondents who receive the information after the outcome is measured as the control group and Figure S6b uses individuals who receive the information treatment for a different behavior as the control group. Table S8 presents results from the distribution regressions of the same analysis. This allows us to understand across which thresholds the treatment has induced people to cross. The coefficients indicate that the treatment is inducing people to report they will at least probably take the vaccine and definitely take the vaccine. Similar regressions restricted to those who report they don't know if they will take the vaccine at baseline are presented in Table S9. Finally, estimates of heterogeneous treatment effects from equation 2 are reported in Tables S11 and S12 and Figure S7.

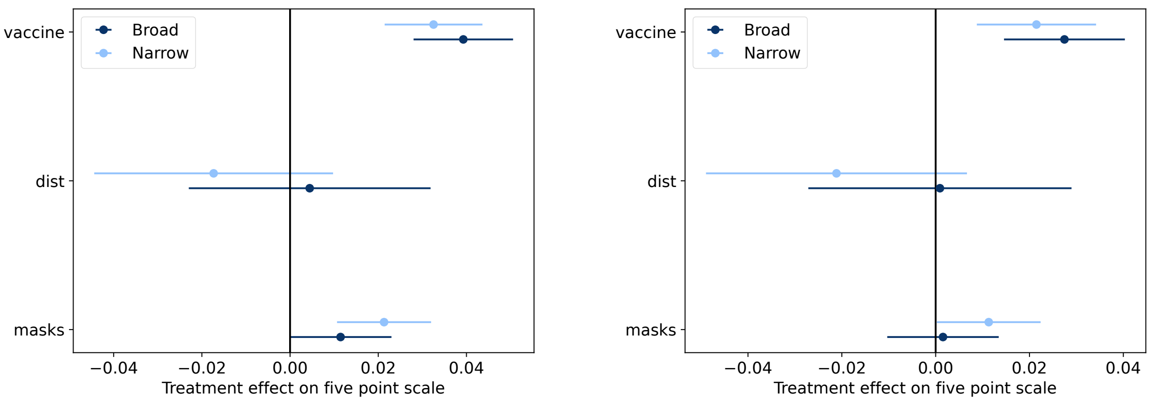

(a) Treatment vs. Control

(b) Treatment vs. Other Behavior Treatment

Fig. S6. Treatment effects with primary and alternative definition of the control group

Graphical illustration of estimates displayed in Table S7. (a) Effect of treatment relative to control group. (b) Effect of treatment relative to treated groups for a different preventative behavior. Error bars are 95% confidence intervals. The exact number of responses are displayed in Table S7.

Table S7. Treatment effects with primary and alternative definition of the control group

	(1)	(2)	(3)	(4)	(5)	(6)
Broad Treatment	0.002	0.001	0.027	0.011	0.004	0.039
	(0.006)	(0.014)	(0.007)	(0.006)	(0.014)	(0.006)
	[-0.010 , 0.013]	[-0.027 , 0.029]	[0.015 , 0.040]	[-0.000 , 0.023]	[-0.023 , 0.032]	[0.028 , 0.051]
	p=0.797	p=0.949	p=2.9E-05	p=0.052	p=0.750	p=8.8E-12
Narrow Treatment	0.011	-0.021	0.021	0.021	-0.017	0.033
	(0.006)	(0.014)	(0.006)	(0.005)	(0.014)	(0.006)
	[0.000 , 0.022]	[-0.049 , 0.007]	[0.009 , 0.034]	[0.011 , 0.032]	[-0.044 , 0.010]	[0.021 , 0.044]
	p=0.045	p=0.136	p=9.2E-04	p=8.9E-05	p=0.210	p=8.8E-09
Control: Other Treatment	X	X	X			
Behavior	masks	dist	vaccine	masks	dist	vaccine
Number Controls	159962	42237	98940	242238	64323	233076
Number Treated	80847	21296	132517	80847	21296	132517
Observations	240,809	63,533	231,457	323,085	85,619	365,593
R^2	0.248	0.234	0.618	0.251	0.244	0.610
Adjusted R^2	0.248	0.233	0.617	0.251	0.243	0.610
Residual Std. Error	0.692	0.855	0.805	0.699	0.856	0.812
F Statistic	143.880	84.485	989.712	204.798	113.443	1513.455

Estimates of equation 1 with intentions as outcomes. All p-values are from two-sided test that coefficient is equal to zero, standard errors are in parentheses, and 95% confidence intervals are in square brackets.

Table S8. Distributional treatment effects

	$>$ No, definitely not	$>$ Probably not	$>$ Unsure	$>$ Probably
Intercept	0.916	0.846	0.673	0.487
	(0.001)	(0.001)	(0.001)	(0.001)
	[0.915 , 0.918]	[0.844 , 0.847]	[0.671 , 0.675]	[0.485 , 0.489]
	p=0.0E+00	p=0.0E+00	p=0.0E+00	p=0.0E+00
Narrow Treatment	0.000	0.005	0.011	0.017
	(0.002)	(0.002)	(0.002)	(0.002)
	[-0.003 , 0.003]	[0.001 , 0.008]	[0.007 , 0.015]	[0.012 , 0.022]
	p=0.824	p=0.009	p=4.3E-07	p=1.4E-11
Broad Treatment	0.000	0.005	0.016	0.019
	(0.002)	(0.002)	(0.002)	(0.002)
	[-0.003 , 0.003]	[0.001 , 0.008]	[0.012 , 0.020]	[0.014 , 0.023]
	p=0.915	p=0.008	p=2.4E-13	p=9.1E-14
Observations	365,593	365,593	365,593	365,593
R^2	0.296	0.493	0.560	0.459
Adjusted R^2	0.296	0.493	0.560	0.459
Residual Std. Error	0.232	0.257	0.310	0.368
F Statistic	152.334	577.685	1647.634	1508.106

Estimates of equation 1 with binary outcomes. The outcome variable for each column is an indicator equal to one if the respondent reported a value higher than the column name. For example, in the column "> Probably not" the outcome Y_i equals one if the respondent answered "Unsure", "Probably", or "Yes, definitely". All p-values are from two-sided test that coefficient is equal to zero, standard errors are in parentheses, and 95% confidence intervals are in square brackets.

Table S9. Distributional treatment effects for "Don't know" respondents

	> No, definitely not	> Probably not	> Unsure	> Probably
Intercept	0.968	0.900	0.295	0.046
	(0.001)	(0.002)	(0.003)	(0.002)
	[0.966 , 0.971]	[0.896 , 0.904]	[0.289 , 0.301]	[0.043 , 0.049]
	p=0.0E+00	p=0.0E+00	p=0.0E+00	p=1.2E-202
Narrow Treatment	0.003	0.005	0.025	0.018
	(0.003)	(0.004)	(0.007)	(0.004)
	[-0.002 , 0.008]	[-0.003 , 0.014]	[0.013 , 0.038]	[0.011 , 0.026]
	p=0.225	p=0.190	p=1.1E-04	p=2.0E-06
Broad Treatment	0.001	0.006	0.049	0.015
	(0.003)	(0.004)	(0.007)	(0.004)
	[-0.004 , 0.006]	[-0.002 , 0.015]	[0.036 , 0.061]	[0.008 , 0.022]
	p=0.732	p=0.126	p=1.9E-13	p=4.5E-05
Observations	69,497	69,497	69,497	69,497
R^2	0.111	0.079	0.091	0.063
Adjusted R^2	0.109	0.077	0.089	0.061
Residual Std. Error	0.167	0.290	0.448	0.218
F Statistic	6.337	8.683	24.288	8.521

Estimates of equation 1 with binary outcomes on sample of respondents who say they don't know if they will take a vaccine at baseline. The outcome variable for each column is an indicator equal to one if the respondent reported a value higher than the column name. For example, in the column "> Probably not" the outcome Y_i equals one if the respondent answered "Unsure", "Probably", or "Yes, definitely". All p-values are from two-sided test that coefficient is equal to zero, standard errors are in parentheses, and 95% confidence intervals are in square brackets.

Table S10. Distributional treatment effects for “Under” respondents

	> No, definitely not	> Probably not	> Unsure	> Probably
Intercept	0.876	0.772	0.549	0.358
	(0.002)	(0.003)	(0.003)	(0.003)
	[0.871 , 0.880]	[0.767 , 0.777]	[0.543 , 0.554]	[0.353 , 0.364]
	p=0.0E+00	p=0.0E+00	p=0.0E+00	p=0.0E+00
Narrow Treatment	0.001	0.012	0.019	0.017
	(0.005)	(0.005)	(0.006)	(0.007)
	[-0.008 , 0.010]	[0.002 , 0.022]	[0.007 , 0.031]	[0.004 , 0.030]
	p=0.849	p=0.017	p=0.002	p=0.008
Broad Treatment	-0.001	0.005	0.019	0.020
	(0.005)	(0.005)	(0.006)	(0.006)
	[-0.010 , 0.009]	[-0.005 , 0.016]	[0.007 , 0.031]	[0.008 , 0.032]
	p=0.844	p=0.320	p=0.002	p=0.002
Observations	48,699	48,699	48,699	48,699
R^2	0.357	0.534	0.580	0.472
Adjusted R^2	0.355	0.532	0.578	0.471
Residual Std. Error	0.266	0.287	0.324	0.352
F Statistic	43.277	176.441	374.368	175.635

Estimates of equation 1 with binary outcomes on sample of respondents who have a baseline beliefs about how many people in their community will take a vaccine under the narrow treatment number. The outcome variable for each column is an indicator equal to one if the respondent reported a value higher than the column name. For example, in the column “> Probably not” the outcome Y_i equals one if the respondent answered “Unsure”, “Probably”, or “Yes, definitely”. All p-values are from two-sided test that coefficient is equal to zero, standard errors are in parentheses, and 95% confidence intervals are in square brackets.

Table S11. Heterogeneous Treatment Effects: Baseline Beliefs

	Average	Above	Between	Under
Broad Treatment	0.039	0.023	0.041	0.043
	(0.006)	(0.017)	(0.015)	(0.016)
	[0.028 , 0.051]	[-0.011 , 0.056]	[0.011 , 0.070]	[0.012 , 0.075]
	p=8.8E-12	p=0.192	p=0.006	p=0.007
Narrow Treatment	0.033	0.026	0.037	0.049
	(0.006)	(0.017)	(0.015)	(0.015)
	[0.021 , 0.044]	[-0.006 , 0.058]	[0.007 , 0.067]	[0.019 , 0.079]
	p=8.8E-09	p=0.115	p=0.015	p=0.001
Observations	365,593	30,731	34,008	48,699
R^2	0.610	0.394	0.625	0.649
Adjusted R^2	0.610	0.391	0.623	0.648
Residual Std. Error	0.812	0.799	0.692	0.826
F Statistic	1513.455	43.505	166.301	329.346

The two-sided joint test that the broad and narrow coefficients are equal across groups has a p-value of 0.807, and the two-sided test that the broad (narrow) treatment effects in the Under and Above groups are equal has a p-value of 0.38 (0.31). All p-values are from two-sided test that coefficient is equal to zero, standard errors are in parentheses, and 95% confidence intervals are in square brackets.

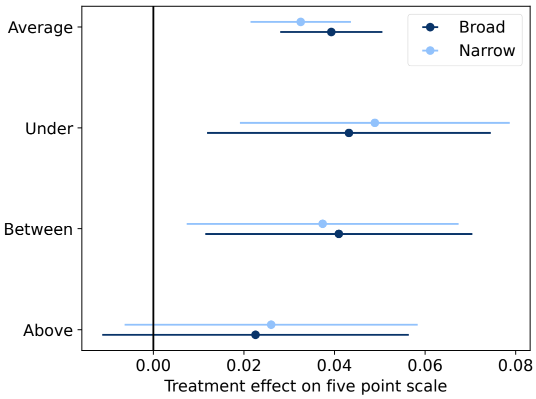

(a) Vaccine Outcome: Baseline Belief Partition

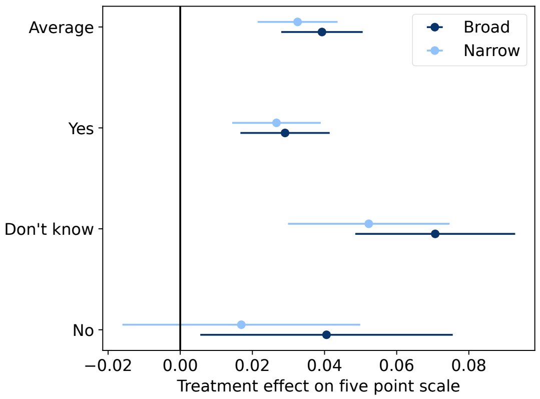

(b) Vaccine Outcome: Vaccine Acceptance

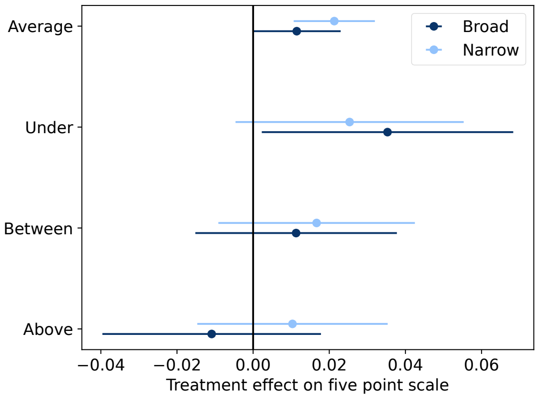

(c) Masks Outcome: Baseline Belief Partition

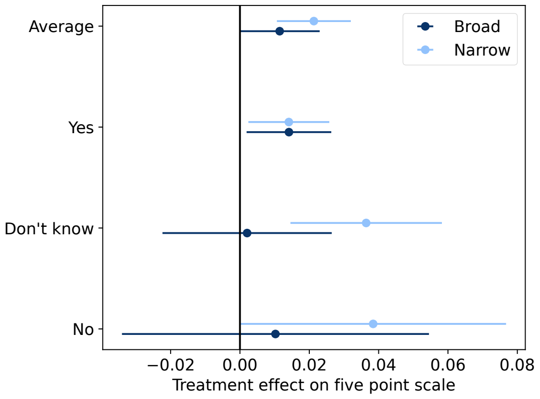

(d) Masks Outcome: Vaccine Acceptance

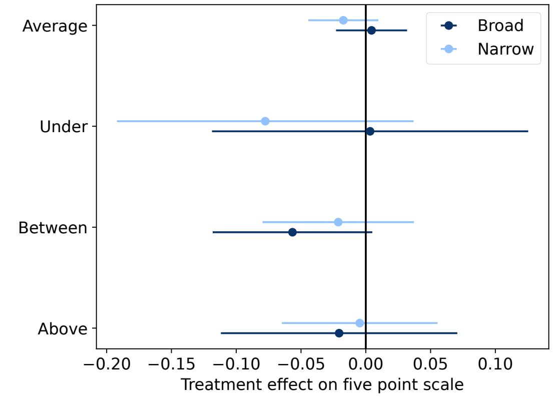

(e) Distancing Outcome: Baseline Belief Partition

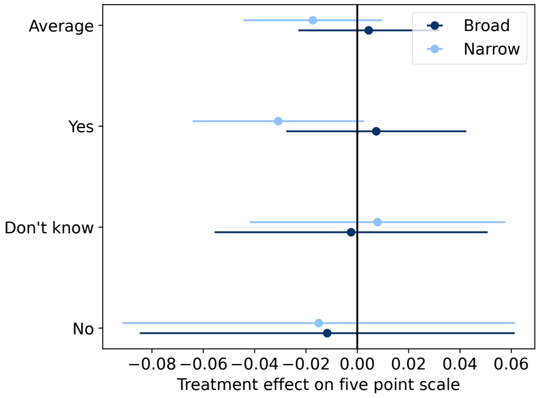

(f) Distancing Outcome: Vaccine Acceptance

Fig. S7. Heterogeneous Treatment Effects

Coefficient on regression of propensity to engage in preventative behavior, treatment, controls, and (centered) treatment control interactions. (a) Heterogeneous effect of treatment on vaccination intentions by baseline belief partition (n=113,438) and (b) baseline vaccine acceptance (n=362,438). (c) Heterogeneous effect of treatment on mask wearing intentions by baseline belief partition (n=102,010) and (d) baseline vaccine acceptance (n=308,550). (e) Heterogeneous effect of treatment on physical distancing intentions by baseline belief partition (n=23,340) and (f) baseline vaccine acceptance (n=84,664). Error bars are 95% confidence intervals.

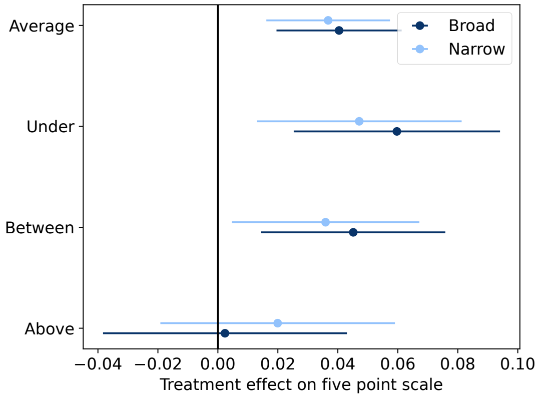

Fig. S8. Mitigating Round Number Bias: Baseline Belief Partition

Heterogeneous treatment effects based on baseline beliefs about how many people in their community will accept a vaccine. We remove respondents who say they believe 0, 50, or 100 percent of people in their community will accept a vaccine to mitigate measurement error due to a bias towards round numbers. Error bars are 95% confidence intervals. There are n=86,617 responses in this analysis.

Table S12. Heterogeneous Treatment Effects: Baseline Vaccine Acceptance

	Average	No	Don't Know	Yes
Broad Treatment	0.039	0.041	0.071	0.029
	(0.006)	(0.018)	(0.011)	(0.006)
	[0.028 , 0.051]	[0.006 , 0.076]	[0.049 , 0.093]	[0.017 , 0.041]
	p=8.8E-12	p=0.023	p=4.1E-10	p=4.2E-06
Narrow Treatment	0.033	0.017	0.052	0.027
	(0.006)	(0.017)	(0.011)	(0.006)
	[0.021 , 0.044]	[-0.016 , 0.050]	[0.030 , 0.075]	[0.014 , 0.039]
	p=8.8E-09	p=0.314	p=4.8E-06	p=2.0E-05
Observations	365,593	53,119	69,497	239,822
R^2	0.610	0.211	0.114	0.119
Adjusted R^2	0.610	0.209	0.112	0.118
Residual Std. Error	0.812	0.994	0.743	0.725
F Statistic	1513.455	50.166	19.873	43.186

The two-sided joint test that the broad and narrow coefficients are equal across groups has a p-value of 0.012, and the two-sided test that the broad (narrow) treatment effects in the Yes and Don't know groups are equal has a p-value of <0.01 (0.05). The two sided test that the broad (narrow) treatment effects in the Don't know and No groups are equal has a p-value of 0.15 (0.08). All p-values are from two-sided test that coefficient is equal to zero, standard errors are in parentheses, and 95% confidence intervals are in square brackets.

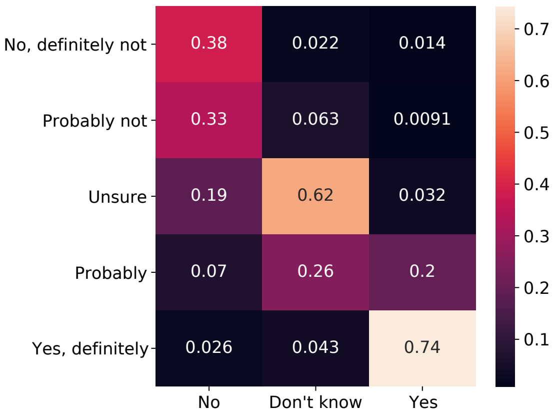

Fig. S9. Correlation of Baseline Vaccine Acceptance and Outcome (Detailed) Vaccine Acceptance

Heatmap showing relationship between baseline vaccine acceptance question (x-axis) and the outcome vaccine acceptance question (y-axis) for the control users. Each cell shows the probability of an outcome response conditional on the baseline response and each column sums to one.

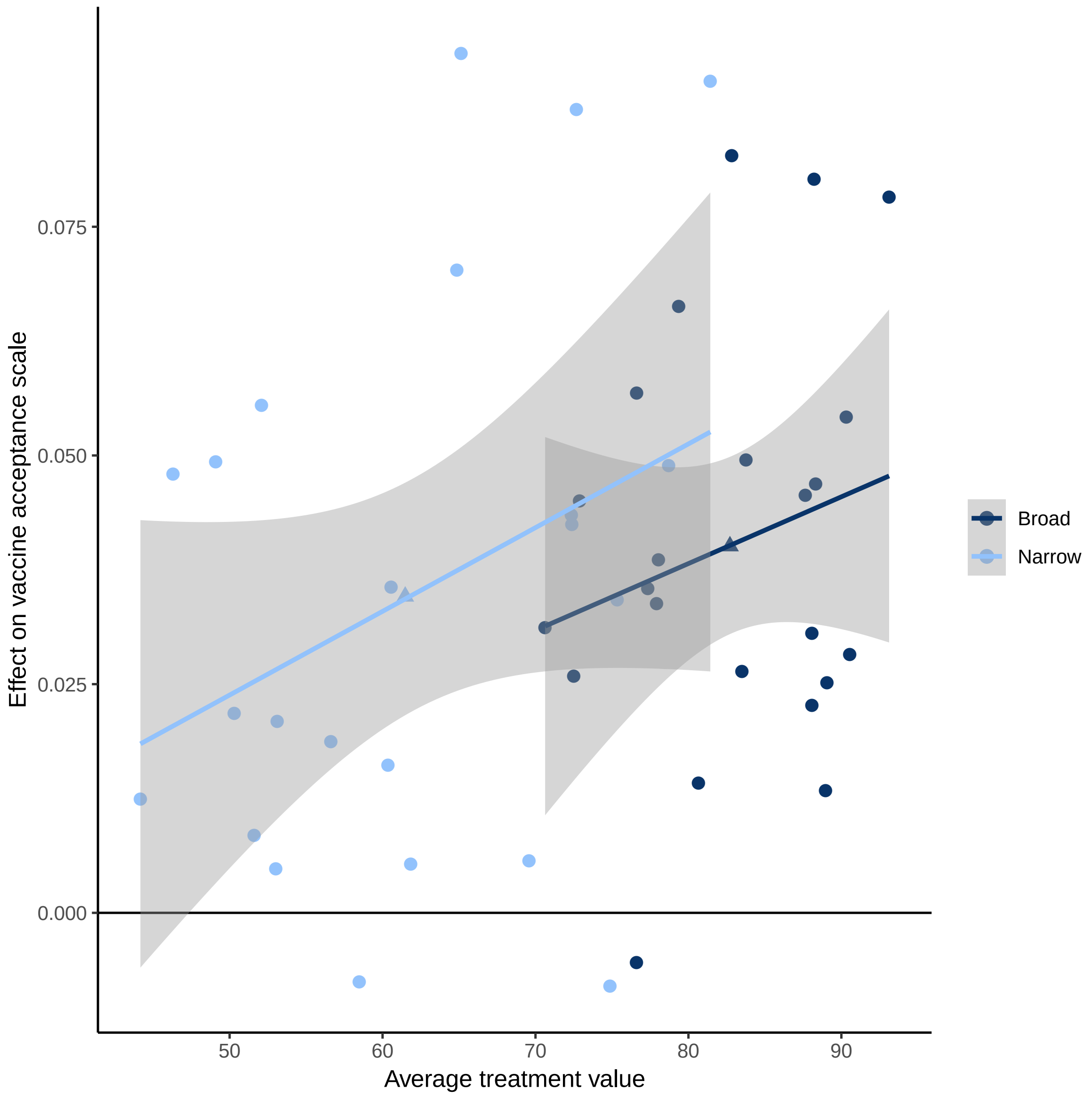

Fig. S10. Explaining Country-Level Heterogeneity with Variation in Treatment Values

A scatter plot of country-level treatment effect estimates and the average normative information treatment shown over the course of the experiment. Each point represents a country-level treatment effect while the triangle represents the grand-mean of treatment effects and the average information shown across all countries in the experiment (weighted by the number of responses). Error bands are 95% confidence intervals.

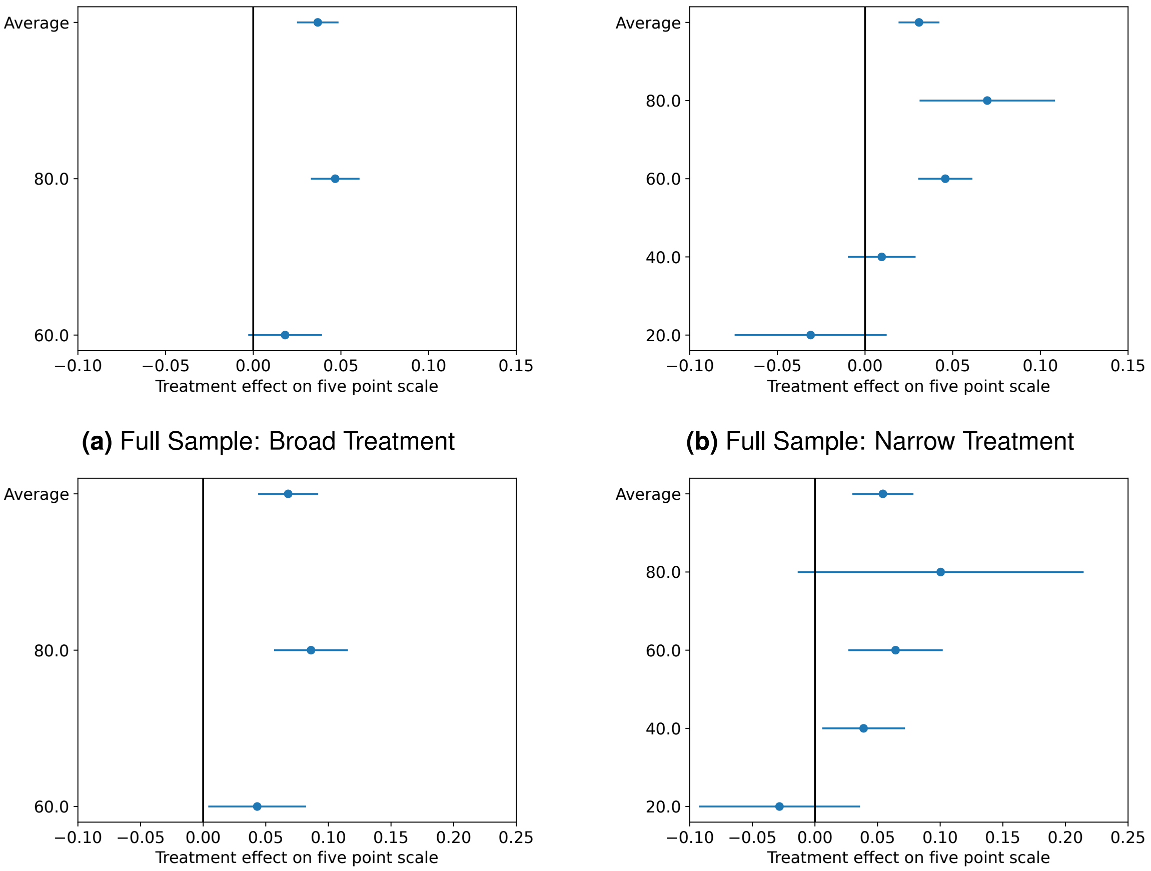

(a) Full Sample: Broad Treatment

(b) Full Sample: Narrow Treatment

(c) Baseline Vaccine Unsure: Broad Treatment

(d) Baseline Vaccine Unsure: Narrow Treatment

Fig. S11. Treatment Effect by Treatment Number

The coefficients reported in these figures are from a regression of vaccine acceptance measured on a five point scale on indicators of the treatment number the individual was shown (if treated) grouped into bins of width 20 percentage points. We also include covariates and their interactions with treatment as in the main analysis. We show the analysis for the (a) full sample receiving the broad treatment (n=299,692), (b) full sample receiving the narrow treatment (n=298,977), (c) sample of respondents reporting “Don’t know” to the baseline vaccine acceptance question and received the broad treatment (n=57,079), and (d) narrow treatment (n=57,113). Error bars are 95% confidence intervals.

Supplementary Note 5.1. Robustness checks. A concern with survey experiments is that results could reflect researcher demand effects, where participants respond how they think the researchers would want them to respond. While we cannot rule this out completely, we do not believe this is driving our results (2, 3). In this section we present results of an analysis that restricts the sample to be separated by at least one "block" of questions between them (Figure S12 and Table S13). Table S14 shows heterogeneous treatment effects by baseline vaccine acceptance for this restricted sample.

Figure S13 plots the distribution of the number of screens between treated and control. In Figure S13a, we plot the distribution for the entire sample and in Figure S13b we plot the distribution for the subset of those with at least one block between treatment and control. For this group there are at least three pages between the treatment and outcome

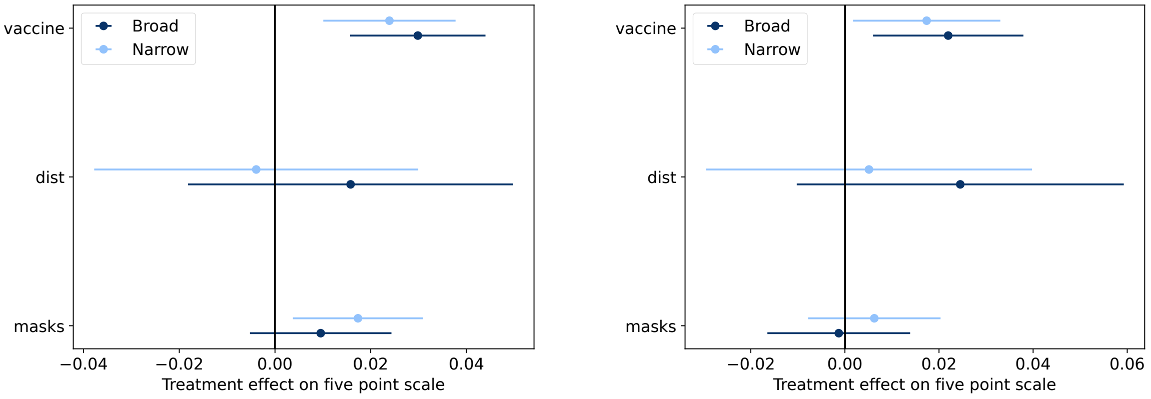

(a) Treatment vs. Control

(b) Treatment vs. Other Behavior Treatment

Fig. S12. Robustness to Researcher Demand Effects

Main analysis conducted on the subset of individuals with at least one "block" of questions separating treatment and outcome. (a) Effect of treatment relative to control group. (b) Effect of treatment relative to treated groups for a different preventative behavior. Error bars are 95% confidence intervals. This is a graphical display of Table S13 and the exact number of observations per analysis are shown there.

Table S13. Robustness to Greater Separation of Treatment and Outcome

	(1)	(2)	(3)	(4)	(5)	(6)
Broad Treatment	-0.001	0.025	0.022	0.010	0.016	0.030
	(0.008)	(0.018)	(0.008)	(0.008)	(0.017)	(0.007)
	[-0.016 , 0.014]	[-0.010 , 0.059]	[0.006 , 0.038]	[-0.005 , 0.024]	[-0.018 , 0.050]	[0.016 , 0.044]
	p=0.866	p=0.167	p=0.007	p=0.205	p=0.362	p=3.5E-05
Narrow Treatment	0.006	0.005	0.017	0.017	-0.004	0.024
	(0.007)	(0.018)	(0.008)	(0.007)	(0.017)	(0.007)
	[-0.008 , 0.020]	[-0.030 , 0.040]	[0.002 , 0.033]	[0.004 , 0.031]	[-0.038 , 0.030]	[0.010 , 0.038]
	p=0.385	p=0.773	p=0.030	p=0.012	p=0.820	p=6.9E-04
Control: Other Treatment	X	X	X			
Behavior	masks	dist	vaccine	masks	dist	vaccine
Number Controls	105973	27441	65464	160843	41734	154676
Number Treated	53773	13784	88139	53773	13784	88139
Observations	159,746	41,225	153,603	214,616	55,518	242,815
R^2	0.222	0.209	0.616	0.226	0.218	0.605
Adjusted R^2	0.221	0.207	0.615	0.226	0.216	0.605
Residual Std. Error	0.707	0.865	0.811	0.715	0.870	0.819
F Statistic	89.371	47.748	652.743	123.872	60.798	980.206

Estimates of equation 1 on the restricted sample when outcome and treatment are separated by at least one additional block of questions. All p-values are from two-sided test that coefficient is equal to zero, standard errors are in parentheses, and 95% confidence intervals are in square brackets.

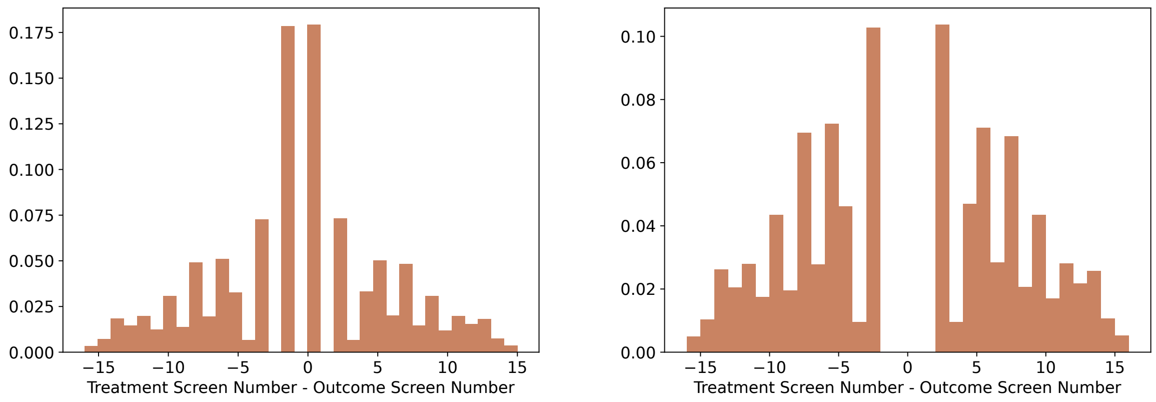

(a) Number of Screens Between Treatment and Outcome

(b) Number of Screens Between Treatment and Outcome in Robustness Check Sample

Fig. S13. Separation of Treatment and Outcome

(a) Histogram of the number of screens between treatment and outcome. Negative numbers represent treated respondents and positive numbers are control respondents. The distribution is not smooth as the randomized order is at the block level, and blocks have varying number of screens (pages) within them. (b) The same histogram, but for the set of respondents with at least one block between treatment and outcome.

Table S14. Robustness to Greater Separation of Treatment and Outcome: Distributional Treatment Effects

	> No, definitely not	> Probably not	> Unsure	> Probably
Intercept	0.916	0.846	0.672	0.486
	(0.001)	(0.001)	(0.001)	(0.002)
	[0.914 , 0.918]	[0.843 , 0.848]	[0.669 , 0.675]	[0.483 , 0.489]
	p=0.0E+00	p=0.0E+00	p=0.0E+00	p=0.0E+00
Narrow Treatment	-0.000	0.002	0.007	0.015
	(0.002)	(0.002)	(0.003)	(0.003)
	[-0.004 , 0.003]	[-0.002 , 0.007]	[0.001 , 0.012]	[0.009 , 0.021]
	p=0.820	p=0.258	p=0.013	p=4.8E-07
Broad Treatment	-0.000	0.002	0.013	0.015
	(0.002)	(0.002)	(0.003)	(0.003)
	[-0.004 , 0.004]	[-0.002 , 0.007]	[0.007 , 0.018]	[0.009 , 0.021]
	p=0.992	p=0.266	p=4.6E-06	p=1.3E-06
Observations	242,815	242,815	242,815	242,815
R^2	0.291	0.490	0.559	0.457
Adjusted R^2	0.291	0.490	0.559	0.457
Residual Std. Error	0.234	0.258	0.312	0.369
F Statistic	100.054	368.874	1071.256	998.540

Estimates of equation 1 on the restricted sample when outcome and treatment are separated by at least one additional block of questions. The outcome variable in this analysis are binary indicators if the outcome was at least a certain response as in table S8. All p-values are from two-sided test that coefficient is equal to zero, standard errors are in parentheses, and 95% confidence intervals are in square brackets.

Supplementary Note 5.2. Unweighted estimates. Here we show the importance of using the survey weights in estimating quantities from the survey (Figure S14a) and the results of the main analyses replicated without using the survey weights (Figure S14b).

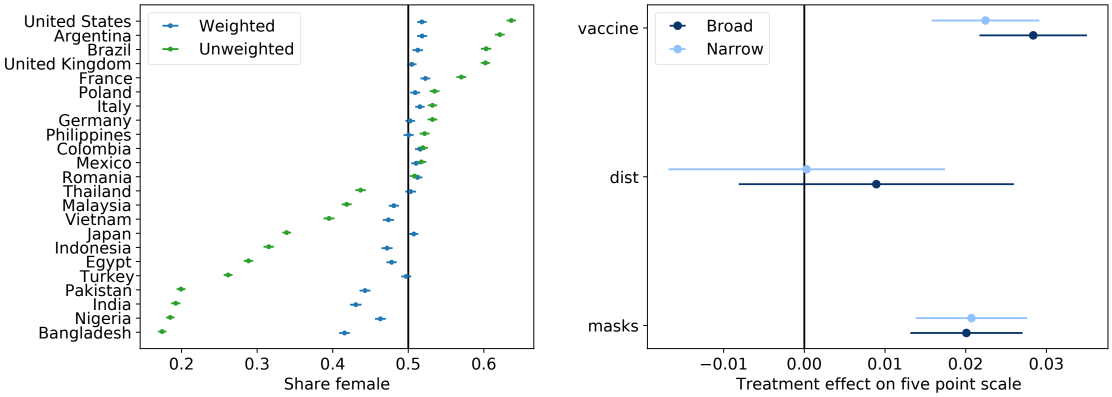

(a) Weighted and unweighted female share

(b) Main effects without weights

Fig. S14. Survey Weights

(a) Weighted and unweighted estimates of the share of females. We expect this to be roughly 0.5, and weights greatly reduce the bias in the unweighted estimates (1, 4). There are n=484,241 responses in this analysis. (b) Main treatment effects using unweighted estimators. There are n=365,593 responses in the vaccine analysis, n=85,619 in the distancing analysis, and n=323,085 in the mask wearing analysis. Error bars are 95% confidence intervals.

Supplementary Note 6. Norm–intention correlations

In the main text, Figure 1 (inset) shows the association between beliefs about descriptive norms and intentions to accept a COVID-19 vaccine. Figure S15 disaggregates this information by country. As in the main text, this is a purely observational association but is computed on the main experimental sample (i.e., starting in late October).

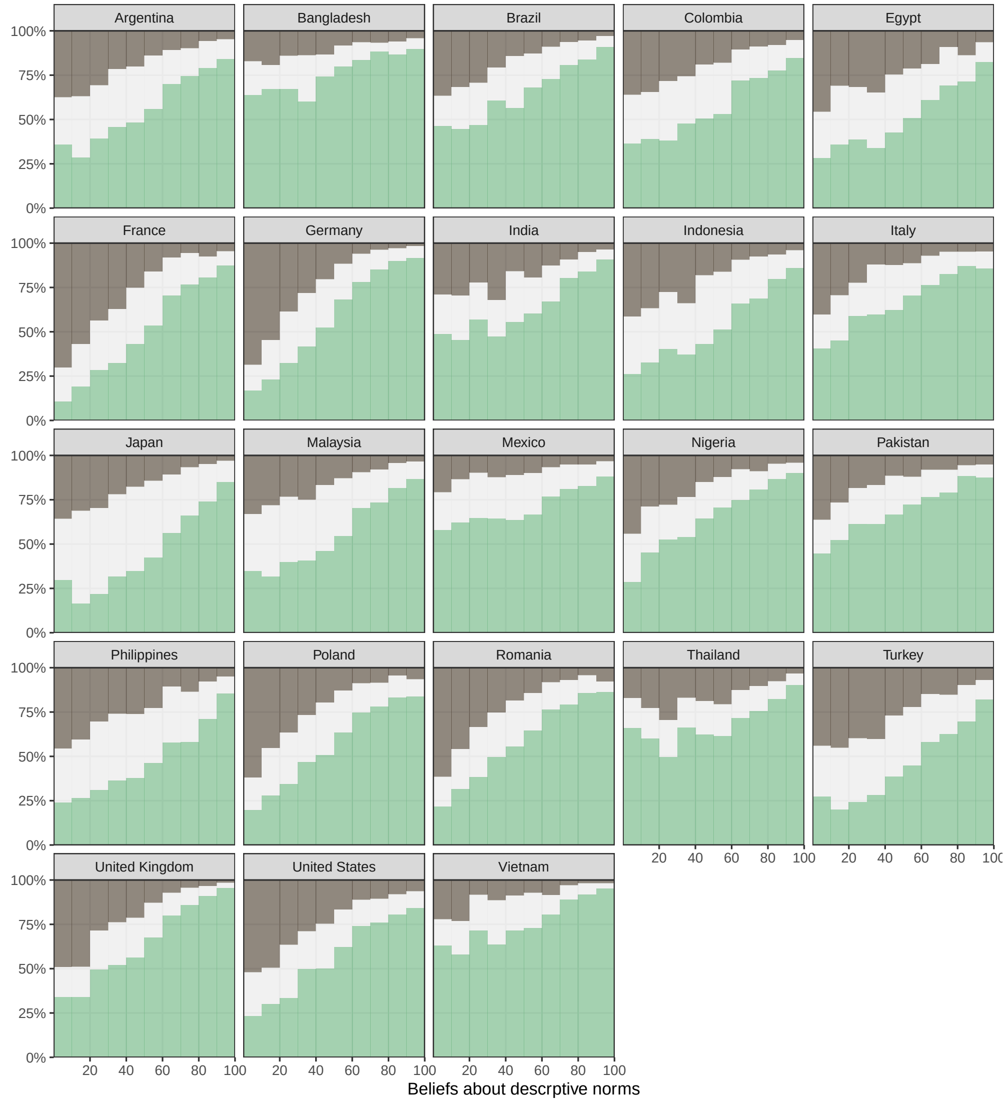

Fig. S15. Correlation in beliefs about norms and intentions by country.

People who believe a larger fraction of their community will accept a vaccine are on average more likely to say they will accept a vaccine and this is true within the 23 included countries. The vertical axis shows the percentage of respondents who replied Yes (green), Don't know (gray) and No (brown) to whether they will accept a COVID-19 vaccine.

Supplementary Note 7. Country-level validation of survey data

At the end of the survey, vaccinations were not readily available to the vast majority of people in the sample and so it is difficult to compare intentions to receive a COVID-19 vaccine with actual vaccine take-up due to supply constraints. In an attempt to quantify the correspondence between our survey responses and other measures of corresponding behavior, we run an auxiliary analysis comparing self-reported receipt of a COVID-19 vaccine from the survey with country-level uptake (COVID-19 vaccine data retrieved from https://github.com/owid/covid-19-data/tree/master/public/data/vaccinations). The estimated vaccinated share of adults from the survey measure is highly predictive of the cross-country variation in actual vaccination shares, explaining over 80% of the cross-sectional variation (Table S15).

Table S15. Survey Data Predictive of Cross-Sectional Vaccine Take-up

	Dependent variable:
Intercept	-1.550
	(1.149)
	[-3.803 , 0.703]
	p=0.177
Survey Share Vacc	0.817
	(0.132)
	[0.559 , 1.075]
	p=5.8E-10
Observations	23
R^2	0.843
Adjusted R^2	0.835
Residual Std. Error	4.354
F Statistic	38.400

Regression of true vaccination share on estimated share vaccinated based on the survey measure. Adjustment for attenuation bias due to measurement error in the survey results in nearly identical results. All p-values are from two-sided test that coefficient is equal to zero, standard errors are in parentheses, and 95% confidence intervals are in square brackets.

Supplementary Note 8. Intention to behavior correlation

A limitation of this experiment is that we measure self-reported vaccination intentions rather than eventual vaccination. When the experiment was first fielded, vaccinations were not available to the public and this remains true for many countries studied throughout the experiment making it difficult or not possible to measure the effect on actual COVID-19 vaccine uptake. To provide some evidence that survey intentions are predictive of vaccination behavior we conducted a supplemental survey in two waves in the United States, where vaccines have become widely available. First, from April 2, 2021 to May 1, 2021 we asked an online panel in the United States from CloudResearch if they had been vaccinated and

their vaccination intentions. We then followed up from May 18, 2021 to June 1, 2021 to ask those who had not been vaccinated at baseline the same question. There were 1,350 respondents who completed both the baseline and endline survey. We then predict endline vaccination status with baseline vaccination intentions and this is plotted inf Figure S16. Our vaccination intentions measure is quite predictive of future vaccination status, with over half of those responding "Yes, definitely" having received at least one dose of a vaccine two months later. We also ask for the approximate date of when they received their vaccine and plot the distribution of acceptance over time in Figure S17, and it is clear that those with stronger intentions to receive a vaccine not only receive the vaccine at higher rates but also do so more quickly.

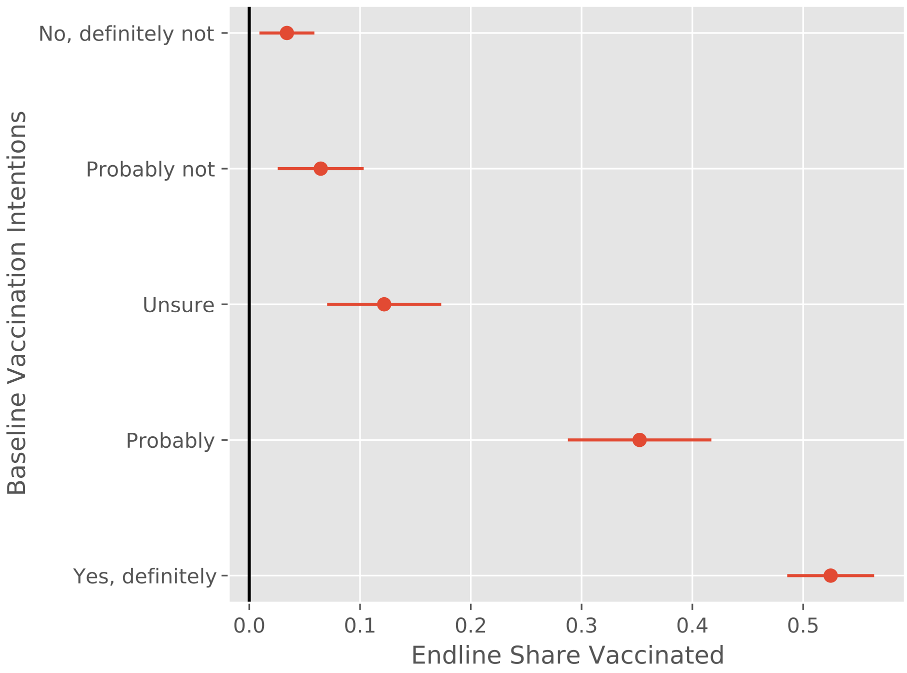

Fig. S16. Vaccination Intentions Predict Future Behavior

Coefficients from regression of endline self-reported vaccination status on baseline vaccination intentions. Error bars are 95% confidence intervals. There are n=1,350 respondents in this analysis.

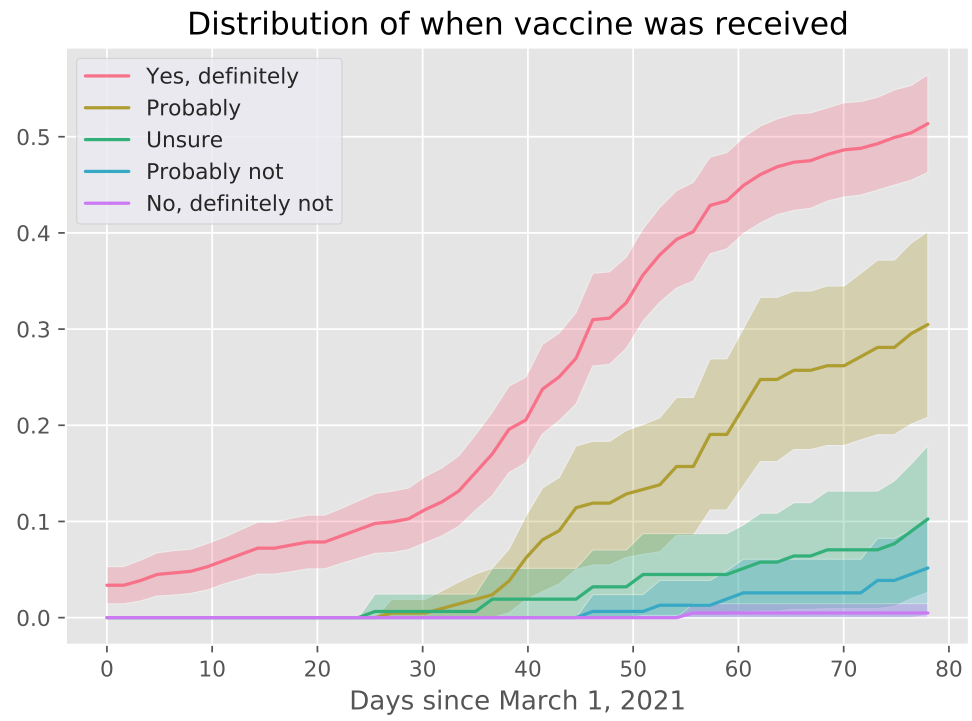

Fig. S17. Vaccination Intentions and Take-up Over Time

Distribution regression of the date that someone received their COVID-19 vaccine by baseline intention group. Those who are unvaccinated at endline are coded as 1,000 so the line plots the share who have received at least one dose over time. There is some mismeasurement, as some respondents reported not having received a vaccine in the baseline survey (April), while saying they received their first dose in March during the endline survey. We plot bootstrapped 95% uniform confidence intervals of the cumulative distribution functions (CDFs) centered around the empirical CDFs. There are n=1,350 respondents in this analysis.

Supplementary References

1. A. Collis, *et al.*, Global survey on covid-19 beliefs, behaviours and norms, *Nature Human Behaviour* pp. 1–8 (2022).

2. J. De Quidt, J. Haushofer, C. Roth, Measuring and bounding experimenter demand, *American Economic Review* **108**, 3266 (2018).

3. J. Mummolo, E. Peterson, Demand effects in survey experiments: An empirical assessment, *American Political Science Review* **113**, 517 (2019).

4. N. Barkay, *et al.*, Weights and methodology brief for the COVID-19 symptom survey by University of Maryland and Carnegie Mellon University, in partnership with Facebook, https://arxiv.org/abs/2009.14675 (2020).
