# Supplementary material for: Providing normative information increases intentions to accept a COVID-19 vaccine
Source: Nat Commun. 2023 Jan 9;14:126. doi: 10.1038/s41467-022-35052-4 (PMC9828376; doi:10.1038/s41467-022-35052-4)
Supplement: Supplementary file 3 — Reporting Summary [file 41467_2022_35052_MOESM3_ESM.pdf]

nature portfolio

Corresponding author(s): Dean Eckles

Last updated by author(s): 14 September 2022

Reporting Summary

Nature Portfolio wishes to improve the reproducibility of the work that we publish. This form provides structure for consistency and transparency in reporting. For further information on Nature Portfolio policies, see our Editorial Policies and the Editorial Policy Checklist.

Statistics

For all statistical analyses, confirm that the following items are present in the figure legend, table legend, main text, or Methods section.

n/a	Confirmed	
☐	☒	The exact sample size (n) for each experimental group/condition, given as a discrete number and unit of measurement
☐	☒	A statement on whether measurements were taken from distinct samples or whether the same sample was measured repeatedly
☐	☒	The statistical test(s) used AND whether they are one- or two-sided<br>*Only common tests should be described solely by name; describe more complex techniques in the Methods section.*
☐	☒	A description of all covariates tested
☐	☒	A description of any assumptions or corrections, such as tests of normality and adjustment for multiple comparisons
☐	☒	A full description of the statistical parameters including central tendency (e.g. means) or other basic estimates (e.g. regression coefficient) AND variation (e.g. standard deviation) or associated estimates of uncertainty (e.g. confidence intervals)
☐	☒	For null hypothesis testing, the test statistic (e.g. F, t, r) with confidence intervals, effect sizes, degrees of freedom and P value noted<br>*Give P values as exact values whenever suitable.*
☒	☐	For Bayesian analysis, information on the choice of priors and Markov chain Monte Carlo settings
☐	☒	For hierarchical and complex designs, identification of the appropriate level for tests and full reporting of outcomes
☐	☒	Estimates of effect sizes (e.g. Cohen's d, Pearson's r), indicating how they were calculated

Our web collection on statistics for biologists contains articles on many of the points above.

Software and code

Policy information about availability of computer code

Data collection	The commercial software service Qualtrics was used for data collection.
Data analysis	All analysis done in python was done using python 3.8 with the following packages numpy (1.21.2), pandas (1.3.0), patsy (0.5.1), scipy (1.6.2), stargazer (0.0.5), statsmodels (0.12.2). The multilevel modeling analysis was run using R version 3.5.1 and additional auxiliary analysis was run using R 4.0.21. Code to replicate the analysis can be found at https://github.com/alexmoehring/SurfacingNormsToIncreaseVaccineAcceptance.

For manuscripts utilizing custom algorithms or software that are central to the research but not yet described in published literature, software must be made available to editors and reviewers. We strongly encourage code deposition in a community repository (e.g. GitHub). See the Nature Portfolio guidelines for submitting code & software for further information.

Data

Policy information about availability of data

All manuscripts must include a data availability statement. This statement should provide the following information, where applicable:

- Accession codes, unique identifiers, or web links for publicly available datasets
- A description of any restrictions on data availability
- For clinical datasets or third party data, please ensure that the statement adheres to our policy

Documentation of the survey instrument and aggregated data from the survey are publicly available at https://covidsurvey.mit.edu. Researchers can request access to the raw (individual level) data from Facebook and MIT at https://dataforgood.fb.com/docs/preventive-health-survey-request-for-data-access/. Moreover, the aggregated data to recreate the figures of this paper have been deposited in https://github.com/alexmoehring/NormsIncreaseVaccineAcceptance (Moehring, 2022) and are provided as Source Data with this paper.

Field-specific reporting

Please select the one below that is the best fit for your research. If you are not sure, read the appropriate sections before making your selection.

- [ ] Life sciences
- [x] Behavioural & social sciences
- [ ] Ecological, evolutionary & environmental sciences

For a reference copy of the document with all sections, see nature.com/documents/nr-reporting-summary-flat.pdf

Behavioural & social sciences study design

All studies must disclose on these points even when the disclosure is negative.

Study description	In a large international survey we introduced a prompt to all respondents that provided normative information about COVID-19 preventative behaviors in their country based on information from the survey. The timing of this information, and the preventative behavior, were randomized allowing us to estimate the causal impact of this information on intentions.
Research sample	All participants were adults and consented to participation in the research via online forms. There were 484,239 participants in the experiment (44% female, modal age group 31-40). There were 1,350 respondents who completed both the initial and follow-up supplemental survey (52% female, average age 40). Subjects in the primary study were not compensated, subjects in the follow-up study were compensated through the online panel CloudResearch.
Sampling strategy	The survey partner Facebook recruited the sample and aimed to achieve a representative sample for the adult population in each country after incorporating the survey weights. The sampling occurred in two-week waves, and Facebook aimed to deliver 3,000 respondents to our survey in each wave.
Data collection	The data were collected using a Qualtrics survey that was distributed by Facebook. Randomization occurred in real-time during the survey and was implemented by Qualtrics. The treatment and hypotheses were not blinded to the researchers.
Timing	The experiment ran from October 2020 until March 2021.
Data exclusions	We required respondents to be eligible for treatment or have a waves survey type (i.e. being in a country with continual data collection). All randomization and balance checks described as ``intent-to-treat'' use this dataset. In our preregistered analysis plan, we described how the sample would be restricted to those who completed the survey and for whom we received a full survey completion weight from Facebook. This removes approximately 40% of respondents.
Non-participation	Roughly 60% of individuals who were eligible for treatment completed the survey and were included in the analysis. In the manuscript we provide robustness checks that were pre-registered to handle differential attrition.
Randomization	We provided the treatment at random times (either before or after the outcome was measured) and the treatment contained information about a randomly chosen preventative behavior. All subjects who were eligible for the information eventually saw the information if they completed the survey.

Reporting for specific materials, systems and methods

We require information from authors about some types of materials, experimental systems and methods used in many studies. Here, indicate whether each material, system or method listed is relevant to your study. If you are not sure if a list item applies to your research, read the appropriate section before selecting a response.

Materials & experimental systems

n/a	Involved in the study	
x		Antibodies
x		Eukaryotic cell lines
x		Palaeontology and archaeology
x		Animals and other organisms
	x	Human research participants
x		Clinical data
x		Dual use research of concern

Methods

n/a	Involved in the study	
x		ChIP-seq
x		Flow cytometry
x		MRI-based neuroimaging

Human research participants

Policy information about studies involving human research participants

Population characteristics	There were 484,239 participants in the primary experiment (44% female, modal age group 31-40). There were 1,350 respondents who completed both the initial and follow-up supplemental survey (52% female, average age 40). Subjects in the primary study were not compensated, subjects in the follow-up study were compensated through the online panel CloudResearch.
Recruitment	Subjects were recruited by Facebook and sent to our platform. In the manuscript we demonstrate substantial sampling and non-response bias that are in large part corrected when survey weights are incorporated. Randomization happens after recruitment, so these biases do not threaten internal validity of the study. Moreover, we show that results are robust to whether or not we adjust using the survey weights.
Ethics oversight	The MIT Committee on the Use of Humans as Experimental Subjects approved both the original survey (protocol E-2294) and the randomized experiment (protocol E-2674) as exempt studies.

Note that full information on the approval of the study protocol must also be provided in the manuscript.
